# TNF induced cleavage of HSP90 by cathepsin D potentiates apoptotic cell death

**DOI:** 10.18632/oncotarget.12411

**Published:** 2016-10-03

**Authors:** Jürgen Fritsch, Ricarda Fickers, Jan Klawitter, Vinzenz Särchen, Philipp Zingler, Dieter Adam, Ottmar Janssen, Eberhard Krause, Stefan Schütze

**Affiliations:** ^1^ Institute of Immunology, Christian-Albrechts-University of Kiel, Kiel, Germany; ^2^ Leibniz Institute for Molecular Pharmacology, Berlin, Germany

**Keywords:** TNF-R1, apoptosis, HSP90, cathepsin D

## Abstract

During apoptosis induction by TNF, the extrinsic and intrinsic apoptosis pathways converge at the lysosomal-mitochondrial interface. Earlier studies showed that the lysosomal aspartic protease Cathepsin D (CtsD) cleaves Bid to tBid, resulting in the amplification of the initial apoptotic cascade via mitochondrial outer membrane permeabilization (MOMP).

The goal of this study was to identify further targets for CtsD that might be involved in activation upon death receptor ligation. Using a proteomics screen, we identified the heat shock protein 90 (HSP90) to be cleaved by CtsD after stimulation of U937 or other cell lines with TNF, FasL and TRAIL. HSP90 cleavage corresponded to apoptosis sensitivity of the cell lines to the different stimuli. After mutation of the cleavage site, HSP90 partially prevented apoptosis induction in U937 and Jurkat cells. Overexpression of the cleavage fragments in U937 and Jurkat cells showed no effect on apoptosis, excluding a direct pro-apoptotic function of these fragments. Pharmacological inhibition of HSP90 with 17AAG boosted ligand mediated apoptosis by enhancing Bid cleavage and caspase-9 activation. Together, we demonstrated that HSP90 plays an anti-apoptotic role in death receptor signalling and that CtsD-mediated cleavage of HSP90 sensitizes cells for apoptosis. These findings identify HSP90 as a potential target for cancer therapy in combination with death ligands (e.g. TNF or TRAIL).

## INTRODUCTION

Tumor necrosis factor alpha (TNF-α) is a pleiotropic cytokine involved in regulating various cellular processes such as inflammation, differentiation, proliferation and apoptosis induction. TNF can trigger signal transduction via two different members of the TNF-receptor superfamily: TNF-receptor 1 (TNF-R1) and TNF-receptor 2 (TNF-R2). TNF-R1 is a member of the death-receptor subgroup of this protein family sharing the so called “death domain” (DD) in their C-terminal domain, which enables them to trigger apoptosis induction [1]. The switch between non-apoptotic and apoptotic signal transduction induced by TNF-R1 depends on the selective recruitment of adaptor proteins to its intracellular part. In fact, we have shown that different adaptor proteins are recruited depending on the subcellular localization and ubiquitination state of the receptor. Plasma membrane resident receptors recruit “complex I” proteins such as TRADD, RIP1, TRAF2 and c-IAP1. Internalized, activated TNF-R1 recruits FADD and caspase-8 to the so called “complex II” and initiaties apoptosis induction [2–6]. Caspase-8 is activated by autoproteolysis and subsequently activates caspase-3 directly or via a lysosomal-mitochondrial amplification loop that involves the release of cytochrome C and APAF-1, and formation of the apoptosome with caspase-9 [7].

Adressing lysosomal-mitochondrial apoptosis amplification in more detail, we found that within TNF-receptosomes, caspase-8 activates caspase-7 which then activates the lipase acid sphingomyelinase (A-SMase) by partial proteolysis in an endo-lysosomal compartment. A-SMase initiates the production of ceramide and activation of Cathepsin D (CtsD) which results in the cleavage of Bid and activation of caspase-3 and -9 [7–10]. However, it still remained unclear whether CtsD exerts other functions in TNF-mediated apoptosis apart from cleaving Bid?

Here, we present a set of putative CtsD substrates that we identified using a differential proteome analysis of CtsD treated and untreated cell lysates. We demonstrate a TNF-induced cleavage of HSP90 by CtsD and identified the respective cleavage sites. Overexpression of exogenously expressed cleavage fragments, however, revealed no boost of apoptosis, while mutation of the cleavage site resulted in partial protection from apoptosis. Inhibition of CtsD by Pepstatin A partially decreases TNF mediated apoptosis induction. In contrast, blocking HSP90 activity by the geldanamycin analogue 17AAG sensitized cells for TNF dependent cell death associated with an increase in Bid cleavage and caspase-9 activity. In addition we showed that the pathway is not restricted to TNF. HSP90 cleavage also occurred after stimulation of various cell lines with FasL and TRAIL.

## RESULTS

### Identification of putative novel CtsD substrates

For the identification of CtsD substrates, we used lysates from CtsD knockout fibroblasts and compared untreated lysates and lysate exposed to exogenously added human liver CtsD by 2D-differential gel electrophoresis. Differences in protein abundance were quantified and differential spots were excised. Proteins were in-gel digested with trypsin and analysed by MALDI mass spectrometry. 8 proteins with a major decrease in protein abundance after *in vitro* CtsD treatment were identified with high confidence (Table 1).

**Table 1:** Displays the stats of the identified proteins

Protein name (murine)	accession number (gi)	vol. ratio	peptides ident.	protein Score	MW	pI
HSP1, beta	40556608	−10,67	6	100	83229,1	4,97
GART	93102415	−4,04	4	100	107435,6	6,25
PP2A	3342500	−5,5	12	100	35611,4	5,3
14-3-3 zeta	1841387	−74,13	11	100	27707,8	4,72
TCP1 theta	5295992	−6,72	3	100	59530,6	5,44
PGK1	70778976	−4,84	6	100	44534	8,02
Dia1	19745150	−4,33	8	100	34105,8	8,55
Ndufs2	2334646	−16,28	17	100	52591,6	6,5

**Figure 1:**
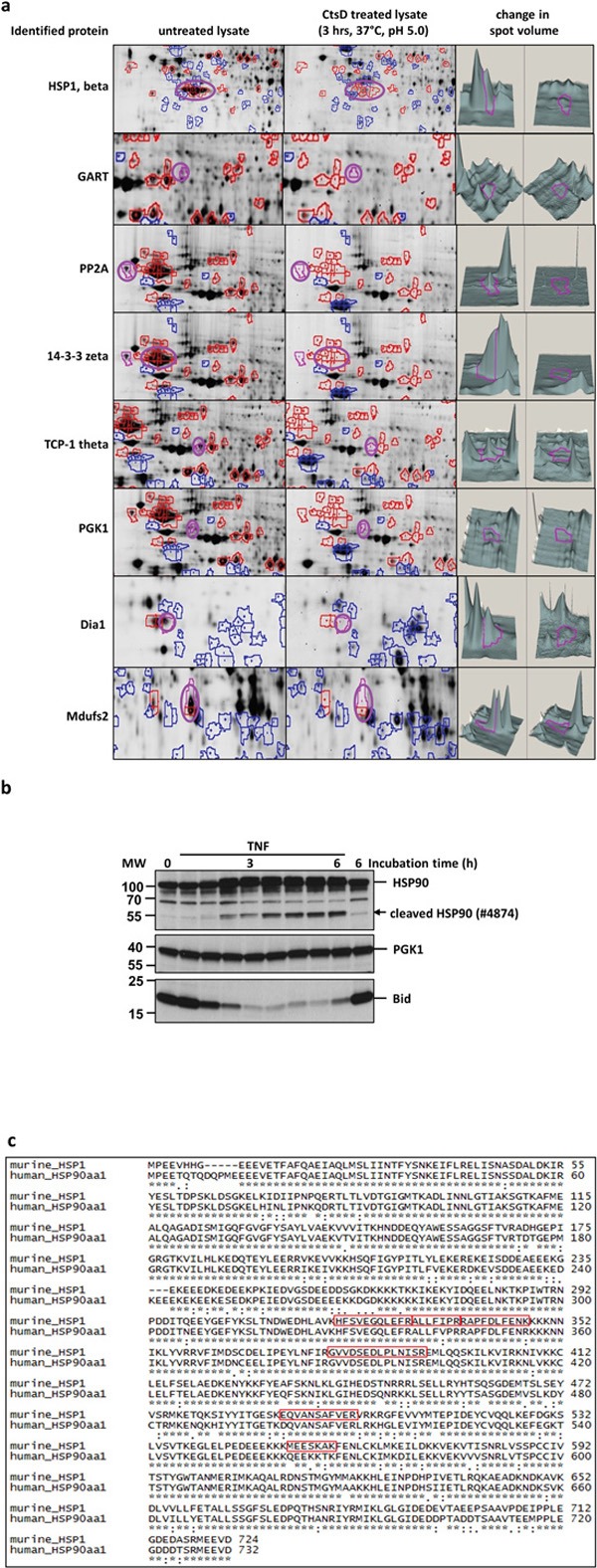
Identification of novel Cathepsin D substrates **a.** Sections from 2D-DIGE gels on which untreated and CtsD treated lysates from CtsD deficient MEF cells had been separated. The first column depicts the name of the protein identified by mass spectrometry. The second column shows present spots (purple circle) from untreated lysates. The third column shows the CtsD treated sample and the reduced spot (purple circle). The fourth column shows the variant spot volumes measured by the DeCyder 6.5 software. **b.** The first Western blot panel shows the HSP90 band pattern after incubating cells up to 6 h with TNF. The appearing cleavage fragment is indicated by the arrow head. PGK1 was also identified by MS in the 2D-DIGE as a putative CtsD substrate but does not respond to TNF in the same time frame (second panel). The TNF and time dependent truncation of Bid in response to TNF is shown the third panel. The data are representative of 3 experiments giving similar results. **c.** Shows the alignment of the identified murine HSP1 and its human homologue HSP90. In the murine sequence, the identified peptides are marked in red squares.

In Figure 1a, the protein identified by mass spectrometry and the in gel localization in untreated and treated samples with a 3D plot of the relevant peaks is shown.

To analyse if the protein band pattern of the identified proteins changes in response to TNF treatment, U937 cells were treated with TNF for up to 6 h. The band pattern of all proteins was analysed by Western blot. We detected a formation of a cleavage product for HSP90 with an onset at 90 min (Figure 1b). Within the same time frame, Bid is degraded. The band pattern of the other proteins (e.g. PGK1) did not change in response to TNF within the given time course.

We therefore focused on the protein HSP1 or rather its human homologue HSP90. As depicted in Figure 1c, both proteins show a high degree of identity and thus, most identified murine peptides (red squares) were also present in the human in the human HSP90.

### TNF induces Cathepsin D catalyzed HSP90 cleavage

**Figure 2:**
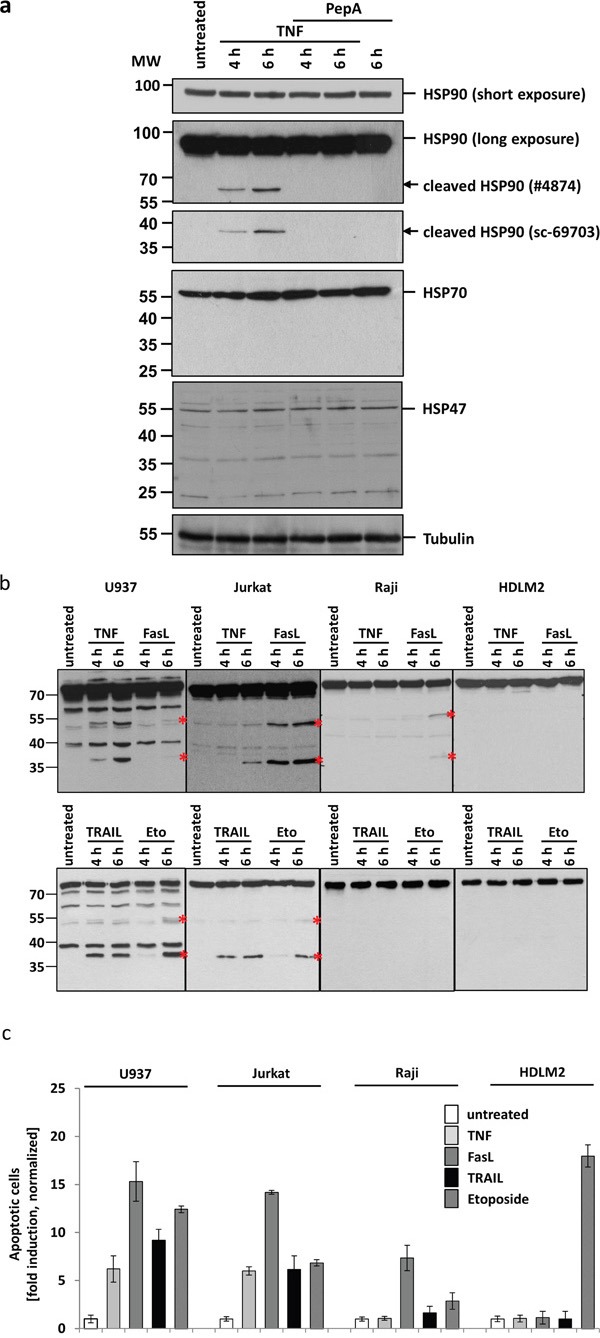
CtsD mediates HSP90 cleavage upon apoptosis induction by TNF **a.** Western blots of untreated and TNF treated U937 lysates: Incubation of cells with TNF for 4 to 6 h results in the appearance of HSP90 cleavage fragments (panel two and three, marked by arrows). The upper cleavage fragment can be detected using antibody #4874 (Cell signalling), the lower fragment using the antibody sc-69703 (Santa Cruz). Pre-treatment of cells with the CtsD inhibitor pepstatin A prevents cleavage fragment formation. The total amount of HSP90, comprising all different HSP90 isoforms, does not decrease (short exposure, panel one). Other HSPs are not affected (panel four: HSP70, panel five: HSP47). Tubulin serves as loading control. **b.** Different cell lines have been treated with TNF, FasL, TRAIL and etoposide to induce apoptosis. Cleavage fragment formation (marked by asterisks) can be observed in U937 and Jurkat as well partially in Raji cells. An example from 3 independent experiments with similar results is shown. **c.** The normalized apoptotic response after towards different stimuli is shown. For quantification, the nuclear fragmentation measured by ImageStream was analysed after 20h incubation with the different ligands. The means of three different independent experiments are shown.

To test whether the TNF induced HSP90 cleavage is CtsD dependent, we treated U937 cells with TNF in the presence of or absence of the CtsD Inhibitor pepstatin A (PepA). Figure 2a demonstrates a time dependent appearance of two HSP90 cleavage products at approx. 60 kDa and 40 kDa. Notably, the two forms were detected with two different antibodies from SantaCruz (40 kDa, sc-69703) and cell signalling (60 kDa #4847), respectively. The formation of these products was not visible in the presence of PepA. The major portion of the HSP90, representing different HSP90 isoforms recognized by the same antibody, is apparently not subject to cleavage under these conditions (short exposure). Moreover, the molecular weight (MW) of the two cleavage fragments suggests a single cleavage. As controls, we also analysed HSP70 and HSP47 which, however, were not altered after TNF treatment.

To analyse whether HSP90 cleavage is TNF-R1-specific or might be a more general phenomenon upon death receptor ligation, we treated different cell lines with TNF, FasL, TRAIL or etoposide as a control. As shown in Figure 2b, TNF treatment results in the HSP90 cleavage only in U937 and Jurkat cells but not in Raji or HDLM2 cells (upper panels). Indeed, FasL induces an even stronger cleavage in Jurkat cells and after 6 h also in Raji cells. TRAIL apparently induces HSP90 cleavage in U937 cells. In HDLM2 cells, no cleavage was seen after any of the stimuli. Longer incubation times have not been analysed.

Figure 2c shows the apoptotic response of the different cell lines towards the death receptor stimuli, measured by nuclear fragmentation analysis using an ImageStream device. U937 and Jurkat responded to TNF, FasL and TRAIL after 20 h incubation. Raji cells also showed nuclear fragmentation after FasL stimulation and only mildly after etoposide incubation. HDLM2 cells did not show any response towards ligand induced apoptosis, but responded vigorously to etoposide treatment. Of note, the apoptotic responses parallel the potency of the death receptors (but not etoposide) to induce cleavage of HSP90 (as shown in Figure 2b).

### Identification of the CtsD cleavage site within HSP90

To proof the capability of CtsD to cleave HSP90, we again incubated recombinantly expressed HSP90 with purified human liver CtsD *in vitro*, and analysed the resulting fragment pattern by SDS-PAGE followed by SYPRO ruby (supplementary Figure S1a). Native HSP90 migrated at around 90-100 kDa (left lane). When treated with human CtsD, we detected additional bands migrating between 55-70 kDa and also bands migrating around 40 kDa. Notably, CtsD treated HSP90 appeared as a doublet that could have given rise to the doublets of the cleavage products.

In order to identify the CtsD cleavage site, we used the Amino-Terminal Oriented Mass Spectrometry of substrates (ATOMS) protocol described by Doucet and Overall [11] with minimal modification. The list of the N-terminal peptides of HSP90 identified by the ATOMS approach is given in supplementary Figure S1b.

The non-prime sites were matched to the corresponding prime sites identified by MS and a heat map was generated to determine whether the peptides (and their cleavage sites) identified in our experiment were in agreement with the known enzyme specificity of Cathepsin D provided in the MEROPS database (supplementary Figure S1c). In supplementary Figure S1d, all identified peptides (red) and the corresponding potential cleavage sites (green) are marked in the HSP90 amino acid sequence. Based on these findings and given the Western blotting results showing bands at around 40 kDa and 60 kDa appearing after TNF-incubation, we propose two putative TNF-inducible CtsD cleavage sites, at phenylalanine in position 437 or tyrosine in position 465.

### Putative biological role of HSP90 cleavage

At least two different biological functions of a CtsD catalyzed HSP90 cleavage can be envisioned: 1) HSP90 cleavage results in a block of its anti-apoptotic function. 2) The resulting HSP90 c- and n-terminal cleavage fragments themselves exert pro-apoptotic functions.

**Figure 3:**
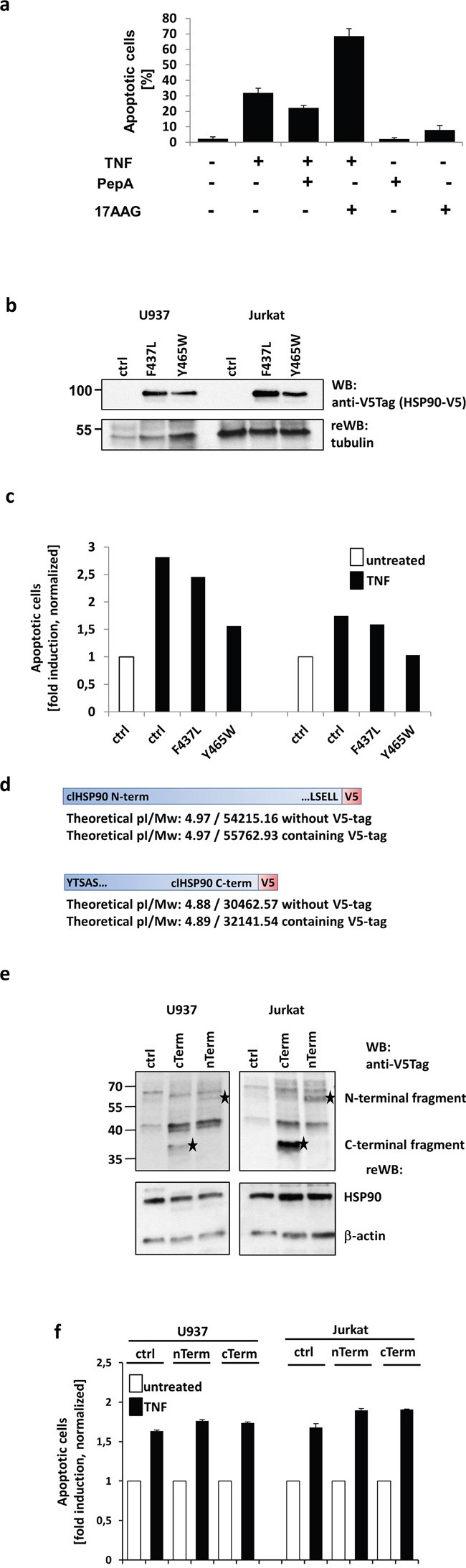
Analysis of the biological outcome of HSP90 cleavage **a.** Apoptosis was measured after 20 h by quantifying nuclear fragmentation by ImageStream analysis: U937 cells were left untreated (~2%) or TNF treated (~30%) and in combination with Pepstatin A (PepA) (~20%) or 17AAG (~70%). PepA and 17AAG showed no cytotoxicity alone. **b.** HSP90-V5 was either mutated at F437 to L or at Y465 to W or then overexpressed in U937 (left) and Jurkat (right) cells. The expression was analysed by Western blot via a V5-tag, tubulin served as loading control. **c.** Overexpression of mutated HSP90 resulted in reduced apoptosis compared to mock transfected cells (ctrl). The Y465W mutant showed higher protective efficiency than the F437L mutant. **d.** Scheme of the molecular mass matched (compared to the Western blots from Figure 2A and B) V5-tagged N- and C-terminal cleavage products. **e.** The expression of the two V5-tagged fragments in U937 (left panels) and Jurkat cells (right panels) is shown and indicated by asterisks. The lower panels show the endogenous HSP90 and actin as loading control. **f.** The normalised apoptotic response towards TNF after transfecting the cells with the V5 tagged constructs or mock plasmid (ctrl) is shown. For quantification, the nuclear fragmentation measured by ImageStream was analysed after 20h incubation with the apoptosis inducing agent.

To address this, we first analyzed the impact of CtsD-inhibition using PepA and HSP90-inhibition using the geldanamycin analogue 17AAG (Tanespimycin) on TNF induced apoptosis. TNF treatment alone results in about 30% apoptotic U937 cells after 18 h of treatment compared to untreated cells (Figure 3a). When cells were pre-incubated U937 cells with the CtsD inhibitor pepstatin A, we observed a partial (~10%) protection from apoptosis. In contrast, preincubation in presence of the HSP90 inhibitor 17AAG significantly increased apoptotic cells compared to TNF treatment alone. This indicated that HSP90 has a strong cytoprotective, anti-apoptotic activity. Similar results were obtained in Jurkat cells (Supplementary Figure S2a). However, 17AAG pretreatment could not sensitize TNF resistant Raji and HDLM2 cells to TNF-induced apoptosis. In addition, when U937, Jurkat, Raji and HDLM2 cells were pretreated with 17AAG followed by incubation with FasL or TRAIL for 20 hours, the apoptotic response towards both ligands was not potentiated (Supplementary Figure S2b).

Based on these results, we concluded that HSP90 mediated apoptosis prevention might be TNF-specific. This might probably be explained with critical role of TNF-R1 internalization for the induction of apoptosis, whereas internalization of other death receptors does not seem to be indispensable for apoptosis induction.

To further analyze the role of HSP90 cleavage in TNF induced cell death, we generated two HSP90 mutants (F437L and Y465W) corresponding to the most probable cleavage sites based on the detected molecular weights. The V5-tagged HSP90 variants were transfected to U937 and Jurkat cells and the expression was verified by Western blot (Figure 3b). When we analyzed apoptosis induction, we noticed that overexpression of both variants led to a partial protection from cell death. Here, the Y465W point mutation proved to be slightly more efficient (Figure 3c). We thus conclude that the phenylalanine in position 465 is probably the main target of TNF induced HSP90 cleavage.

To investigate a potential pro-apoptotic function of the resulting c- and n-terminal parts of HSP90, we generated the corresponding fragments with a V5-tag for detection (Figure 3d). The constructs were transfected to U937, Jurkat cells and protein expression was analysed by Western blot (Figure 3e). The C- and N-terminal fragments are marked with an asterisk in the upper blot panels. Whereas the lower panels show HSP90 and actin as loading controls. In parallel C-term and N-term transfected or mock-transfected cells were treated with TNF and apoptosis induction was analysed. As depicted in Figure 3f, we could not observe an increase in apoptosis induction, neither in U937 nor in Jurkat cells.

From these experiments we concluded that CtsD-mediated cleavage interferes with its anti-apoptotic function rather than actively supporting cell death induction by novel pro-apoptotic peptides.

### Analysis of HSP90 downstream signalling

**Figure 4:**
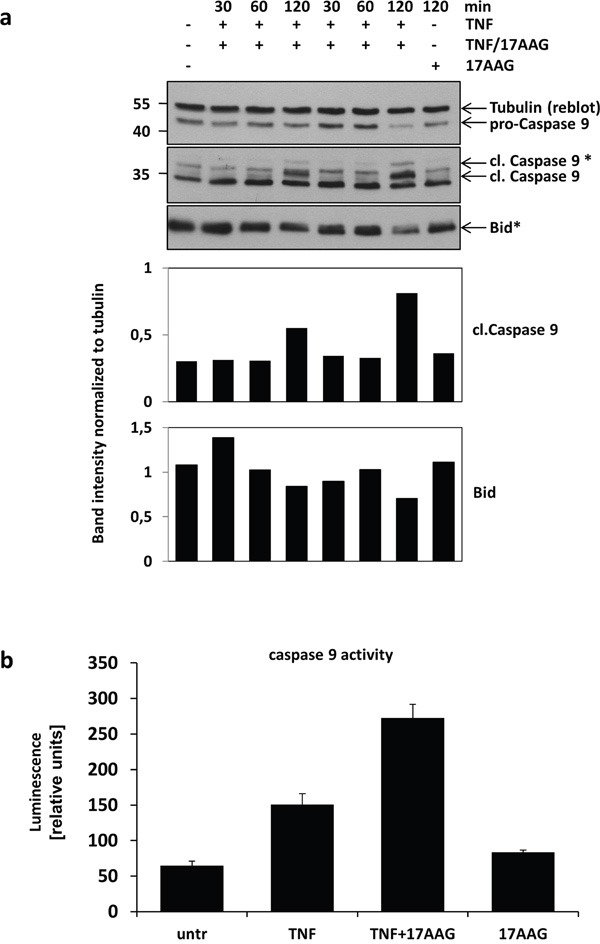
Analysis HSP90 downstream signalling **a.** The impact of TNF treatment in combination with HSP90 inhibition on the activation of caspase 9 and Bid was analysed by Western blot. The blots show the decrease in pro-caspase 9 levels and the increase in cleaved caspase 9 levels after 120 min. After the same time, more Bid-cleavage can be observed. Densitometric quantification of the Western blots is shown below (representative result of three experiments). **b.** Increases in enzymatic activity of caspase 9 after TNF and combined TNF/17AAG treatment compared to untreated cell lysates was analysed by luminescence measurement (n=3).

Although we showed that HSP90 protected cells from apoptosis induction, the putative HSP90 downstream molecules were still enigmatic. Following earlier studies [12, 13], we analysed the activation of caspase 9 and also Bid cleavage in cells treated with TNF alone or in combination with 17AAG. In line with the apoptosis enhancing effect of HSP90 inhibition shown before, we found that inhibition of HSP90 resulted in enhanced Bid and caspase 9 cleavage (Figure 4a) as well as an increase in enzymatic caspase 9 activity (Figure 4b).

## DISCUSSION

**Figure 5:**
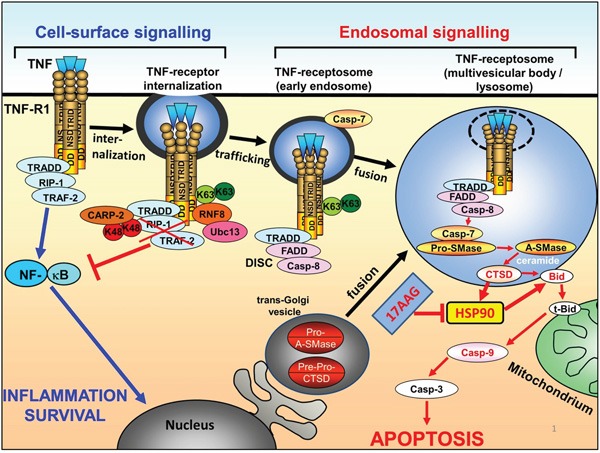
Scheme of HSP90 inactivation on the context of apoptotic TNF-R1 signal transduction Binding of TNF to TNF-R1 initially triggers survival signalling via TRADD/RIP1/TRAF2 and NF-kB activation. K63 ubiquitination of TNF-R1 mediates internalization of the receptor and formation of TNF-receptosomes, concomitantly switching off NF-kB signalling and recruiting the DISC proteins FADD and caspase-8. The TNF-receptosomes maturate by fusing with trans-golgi vesicles to form multivesicular body compartments. Cathepsin D is activated and released into the cytosol, where it can cleave Bid and also HSP90. Cleavage of HSP90 or inhibition of HSP90 by 17AAG finally results in enhanced Bid cleavage, Caspase 9 activation and apoptosis.

Proteolysis is a major hallmark of apoptotic cell death. It is well established that besides caspases, also lysosomal cathepsin proteases are involved in apoptosis signalling. One prominent member is cathepsin D (CtsD). Which is critical for lysosomal membrane permeabilization (LMP), which often serves to amplify the cell death signalling, but can also be an initial trigger [7, 14]. In earlier work, we demonstrated a role for the aspartic protease CtsD in TNF induced apoptosis signalling (see, Figure 5). We showed that after TNF-R1 internalization, which is absolutely mandatory for apoptosis induction, CtsD is activated in the endolysosomal compartment by a proteolytic activity of caspase 8 and 7 which leads to the activation of acid sphingomyelinase and ceramide production inside the endo-lysosomes. Ceramide triggers the autoproteolytic activation of the CtsD zymogen and its translocation from the endolysosomal compartment to the cytosol. In the cytosol, CtsD cleaves Bid to form the pro-apoptotic tBid, that is involved in the release of cytochrome C from the mitochondria [2, 4, 8–10]. The CtsD mediated Bid activation and also its cleavage site was later confirmed by Appelqvist and colleagues [15]. At the stage of mitochondrial outer membrane permeabilization (MOMP), the receptor mediated extrinsic and the intrinsic cell death pathways converge. Besides Bid cleavage, cysteine cathepsins degrade other anti-apoptotic proteins like Bcl-2 proteins and XIAP [14].

Apart from Bid no other apoptosis related cathepsin D substrates are known to date [16]. To identify putative CtsD substrates, lysates from CtsD deficient MEF cells were incubated with purified CtsD, and compared by 2D-DIGE. Moreover, changes in the abundance or band patterns of the respective proteins were analysed in response to TNF treatment for (4-6 h). Following HSP90, we observed the formation of stable cleavage fragments of HSP90. Pre-incubation with the CtsD inhibitor pepstatin A prevented TNF-induced HSP90 cleavage. After identification of the putative cleavage sites using a modified ATOMS approach, we overexpressed HSP90 peptides and found that these “neo-proteins” do not display pro-apoptotic capacity. Point mutations of the most probable cleavage sites F437 and Y465 and overexpression of these constructs resulted in a partial protection from TNF-induced apoptosis. Additionally, we showed that HSP90 cleavage does not only occur in response to TNF treatment but also after treatment with FasL and TRAIL and, to a minor extend, after incubating cells with etoposide. Blocking HSP90 using the geldanamycin analogue 17AAG resulted in doubling the apoptotic response towards TNF in cells that were already sensitive. However, it was not possible to overcome TNF-resistance. FasL and TRAIL mediated apoptosis induction was not affected by 17AAG. Taken together, our results show a protective role of HSP90 towards extrinsic TNF mediated apoptosis stimuli in several hematopoietic cell lines.

We observed that stimulation with FasL can also induce HSP90 cleavage while pre-incubation with 17AAG did not result in enhanced apoptosis. Chen et al showed that Caspase 10 can cleave HSP90 in response to UVB mediated FasL secretion and apoptosis induction [17]. Interestingly, the resulting fragment migrated at ~50kDa, a molecular weight similar to the upper band that we detected in our assays. For FasL mediated apoptosis, receptor internalization is not mandatory. Here, the rapid and massive DISC formation at the receptor might be sufficient to also cleave HSP90 and apoptosis amplification via the lysosomal-mitochondrial loop is not necessary. The apoptotic response to FasL is usually much stronger compared to TNF. Thus, HSP90 inhibition by FasL might not contribute to further enhancement of apoptosis induction. This could be similar for TRAIL. For both receptors the need for internalization to induce cell death seems to be cell line and receptor dependent and is still controversially discussed [18–22].

Two studies showed that HSP90 inhibition by radicicol or NVP-AUY922 in combination with TRAIL lead to enhanced apoptosis in colorectal cancer and ovarian carcinoma cells [23, 24]. Zhang and colleagues reported that Celastrol, in contrast to 17AAG sensitized Panc-1 cells to apoptosis induction [25]. We also used Celastrol to inhibit HSP90 interaction with its co-chaperone cdc37 and obtained similar results as for 17AAG (Data not shown). Thus, it might be interesting to broaden the analyses to further HSP90 inhibitors having a different mode of action. It will also be challenging to further analyse the HSP90 cleavage in response to FasL and receptor specific TRAIL variants in the absence or presence of pepstatin A to see if other proteases apart from CtsD are in involved in HSP90 cleavage in these receptor systems. Further it should be investigated if the receptors CD95 and TRAIL-R1 and -R2 have to be internalized in the responding cell lines to mediate HSP90 cleavage.

Beck and colleagues reported on a HSP90 cleavage depending on reactive oxygen species, resulting in a cleavage fragment migrating around 70 kDa [26]. Redlak and Miller reported HSP90 cleavage upon deoxycholate treatment [27]. Moreover, exposure of cells to H_2_O_2_ or other reactive oxygen species might also induce HSP90 cleavage [28, 29]. Rodina and colleagues showed that HSP90 inhibits the intrinsic apoptotic pathway in small-cell lung cancer [30]. Thus, degradation of HSP90 seems to be common in cell death signalling to impede its cytoprotective function.

Observations by Jäättelä and colleagues showed that HSP70 protects cells from lysosomal membrane permeabilization (LMP) by regulating A-SMase activity [31–33]. This prompted us to analyse enriched lysosomal fractions for the occurrence of HSP90 and its cleavage fragments. Moreover, we could not detect HSP90 in these fractions and therefore, concluded that HSP90 is cleaved in the cytoplasm by CtsD released from lysosomes. Therefore, the cleaved HSP90 might have other functions than stabilizing lysosomes as HSP70.

Besides the cleavage of HSP90, which seems to be a relatively late event in TNF-induced apoptosis induction, Vanden Berghe and colleagues showed that blocking HSP90 induces apoptosis instead of necrosis in L929sA cells [34]. This is in line with several recent reports in which for instance Zhao and colleagues showed that functional HSP90 is needed for TNF-induced necroptosis induction by preventing proteasomal degradation of MLKL and RIP3 [35]. Li and colleagues showed that inhibition of HSP90 using kongensin A sensitizes various cell lines for apoptosis rather than necroptosis although independent of death ligands [36] [37].

It was shown that HSP90 can bind to RIP1 and prevent its binding to the TNF-R1. Thus, cleavage of HSP90 could also be a means to amplify TNF-induced pro-apoptotic signalling by releasing more RIP1, which in turn can bind to the activated TNF-R1 [38]. In the present study, however, we only analysed TNF induced apoptotic signalling but not necroptosis induction. It will be interesting to investigate for necroptosis induction in cell lines (Raji and HDLM-2) that were resistant to TNF and TNF/17AAG with regard to apoptosis.

In conclusion, we identified several putative CtsD substrates. One of these proteins is HSP90, which is cleaved upon TNF stimulation in the observed cell lines. Interestingly, most of the other proteins that have been identified showed no alteration upon TNF treatment. Therefore, it remains to be elucidated if these are bona fide CtsD substrates in a cellular signalling context, or if they have just been degraded because they were accessible to CtsD in total cell lysates.

Following earlier studies highlighting the importance of HSP90 cleavage, we now show that Cathepsin D is the responsible protease for HSP90 cleavage in the TNF-induced apoptosis pathway. The capability of HSP90 inhibition to boost TNF-induced apoptosis indicates its potential as a target for a putative combination therapy, which is proven by its application in various clinical trials [39–48]. As a perspective, it will be interesting to see if HSP90 inhibition in combination with death ligands can trigger apoptosis in other tumor entities.

## MATERIALS AND METHODS

### Reagents and antibodies

17AAG was purchased from Tocris. Pepstatin A was purchased from Sigma-Aldrich. HSP90 was purchased from NOVUS biologicals/bio-techne. CtsD was purchased from Loxo. The plasmid coding for Fc-tagged TNF was kindly provided by Harald Wajant, (Julius-Maximilians-University, University Hospital, Würzburg, Germany). FcTNF and FcFasL were expressed in HEK293T cells and subsequently purified using HiTrap Protein G columns (GE Healthcare). KillerTRAIL was purchased from Enzo. MS grade trypsin was from Promega, solvents for LC-MS analysis (acetonitrile, trifluoroacetic acid) and for reductive demethylation (light and heavy formaldehyde) and sodium cyanoborohydride were purchased from Sigma Aldrich.

Primary antibodies used in this study: HSP90 antibodies were from were from Santa Cruz Biotechnology (sc-69703) and from Cell Signaling (#4874). HSP70 (sc-24) and HSP47 (sc-8352) antibodies were from Santa Cruz Biotechnology. Caspase 9 (#7237) antibody was from Cell Signaling. HRP-conjugated anti-tubulin antibody (HRP-66031) was from Proteintech. Anti-V5 (R960-25) antibody was from Life Technlogies. Bid (sc-11423) antibody was from Santa Cruz Biotechnology.

Secondary antibodies used in this study: anti-mouse light chain HRP conjugated (AP200P) and anti-rabbit light chain HRP conjugated (MAB201P) from Millipore.

### Cell culture

Suspension cells (U937, Jurkat, Raji, HDLM-2) were cultured in RPMI 1640 supplemented with 5% FCS (Biochrom) and penicillin / streptomycin (Biochrom). CtsD deficient MEFs were cultured using DMEM supplemented with 10% FCS and penicillin/streptomycin.

### *In vitro* CtsD treatment

Lysates from CtsD deficient MEF cells were prepared in 40mM HEPES pH 6.0, 150mM KCl, 0.5% NP-40. 500 μg of the lysate was incubated with 100 ng of purified human liver CtsD (Loxo) for 5 h at 37°C.

### 2D-DIGE and MS workflow

The 2D-DIGE-, image analysis and the following MS analysis were essentially performed as described in [49]. Prior to sample labelling for 2D-DIGE the desired amount of protein was precipitated in 10% TCA and the precipitate resuspended in labeling buffer containing 7M urea, 2 M thiourea, 30 mM TRIS and 4% CHAPS.

### Protein identification by MALDI mass spectrometry

Tryptic in-gel digestion and sample preparation was performed as previously described [50]. In brief, excised gel spots were incubated with 20 μl 50 mM ammonium bicarbonate containing 50 ng trypsin (sequencing grade modified, Promega) at 37°C overnight, the enzymatic reaction was terminated by addition of 20 μl trifluoroacetic acid in acetonitrile. MS and MS/MS measurements were performed using a MALDI-TOF-TOF instrument (AB SCIEX TOF/TOF 5800; Applied Biosystems, Framingham, MA, USA) equipped with a Neodymium-doped yttrium lithium fluoride laser (Nd:YLF, 349 nm). MS spectra were acquired in positive ion reflector mode by accumulating 5000 consecutive laser shots. For MS/MS, a maximum of 20 precursor ions were selected automatically. GPS Explorer (version 3.6, Applied Biosystems) was used to process the spectra. The processed MS data were searched in-house against the NCBI database (version 02022009) using a MASCOT server (version 2.2.2, Matrix Science Ltd, London). The mass tolerance of precursor and sequence ions was set to 100 ppm and 0.35 Da, respectively. A maximum of two missed cleavages was allowed. Methionine oxidation and the acrylamide modification of cysteine were used as variable modifications. A protein was accepted as identified if the total MASCOT score was greater than the significance threshold and at least two peptides appeared the first time in the report and were the top ranking peptides.

### SDS-PAGE and western blotting

For SDS-PAGE 12.5% PAA gels were used. Proteins were blotted to PVDF membrane (Carl-Roth). The membranes were blocked with 5% skimmed milk in TBST and incubated over night with the primary antibody diluted 1:500-1:5,000 in 5% skimmed milk. The peroxidase conjugated secondary antibodies were incubated for 1 h diluted 1:10,000 in 5% skimmed milk. Blots were developed using the ECL kit and films from GE Healthcare. Bands were scanned using a personal densitometer (GE Healthcare).

### Molecular biology

The genes encoding the V5-tagged HSP90 cleavage fragments and the V5-tagged HSP90 constructs were purchased from Geneart. Cloning into pcDNA3.1/Zeo was performed in house. Sequence validation by Sanger sequencing was performed at the Institute of Clinical Molecular Biology, University of Kiel.

Cells were transfected by electroporation (BioRad Gene Pulser, Jurkat settings).

### Caspase assay

For measuring Caspase activity, the Caspase-Glo® assay from Promega was used according to the manufacturer's recommendations.

### Image stream

For apoptosis measurement cells were incubated for the times indicated in the figure with the respective death ligand (100 ng/ml) under standard cell culture conditions. 30 min before end Hoechst stain (Sigma-Aldrich) was added to the culture medium finally diluted 1:10,000. Up to 10,000 cells were captured, detecting the nuclear stain (excitation: 405 nm) at Channel 1. For the image acquisition the 60x objective was used. The apoptosis wizard was used for assaying the number of cells showing nuclear fragmentation compared to cells with intact nuclei.

## SUPPLEMENTARY DATA AND FIGURES


