# Supplementary material for: TNF induced cleavage of HSP90 by cathepsin D potentiates apoptotic cell death
Source: Oncotarget. 2016 Oct 3;7(46):75774–89. doi: 10.18632/oncotarget.12411 (PMC5342777; doi:10.18632/oncotarget.12411)
Supplement: Supplementary file 1 [file oncotarget-07-75774-s001.pdf]

TNF induced cleavage of HSP90 by cathepsin D potentiates apoptotic cell death

SUPPLEMENTARY DATA

SUPPLEMENTARY METHODS

Amino-Terminal Oriented Mass Spectrometry of substrates (ATOMS)

Briefly, 5 µg of HSP90 (Novus biologicals/bio-techne) was treated either with or without Cathepsin D (Loxo). The reaction was quenched by heating the samples (95 °C, 10 min). Proteins were denatured and reduced using 4M Guanidine HCl in 100 mM HEPES (pH 8.0) with DTT (5mM) for 1 h at 60 °C. Free Cysteine sulfhydryls were alkylated at room temperature for 2 h in the dark with 15mM iodoacetamide (IAA). Next the amine groups (lysine side chains and N-termini) of the protease treated sample were labeled by reductive demethylation using heavy formaldehyde while the amine groups in the control aliquot are labeled with light formaldehyde using 100 mM formaldehyde and 50 mM sodium cyanoborohydride for 16 h at room temperature. Unreacted excess of formaldehyde was quenched using ammonium bicarbonate (ABC, 200 mM) for 2 h. Samples were then combined and acetone precipitated overnight at -20 °C. Sample pellets were redissolved in minimal volume of 4M Guanidine HCl and then diluted using 20 mM ABC buffer to a final Guanidine HCl concentration of 0.5 M. Trypsin was added at a protein to enzyme ratio of 50:1, digestion was performed overnight at 37 °C. Because the lysine residues are dimethylated trypsin mainly cleaves after arginine. Therefore, half of the samples was further digested with Glu-C overnight at a 20:1 (protein:enzyme). Samples were acidified to 0.25% TFA (pH <3) and analysed via LC-MS/MS. LC-MS analysis was performed on a U3000 (Dionex) coupled online to an Orbitrap Velos mass spectrometer (Thermo). MS spectra (300-2000 *m/z*) were acquired with a resolution of 60 000. Data dependent acquisition was performed on the three most intense peaks using both collision induced dissociation (CID) and higer collisional energy dissociation (HCD). Three technical (injection) replicates were performed for each digest.

A second biological experiment was also performed. In this case the control sample was labeled with heavy formaldehyde while the experimental sample was labeled with light formaldehyde to factor in any potential bias with the different (light and heavy) formaldehyde reagents.

ATOMS data processing

All samples were recalibrated offline against the plasticizer polydimethylcyclosiloxane (m/z 445.120025) to increase the mass accuracy of measurements. The reprocessed data were then searched against the entire reviewed canonical Human database with common contaminants (cRAP, http://www.thegpm.org/crap/) appended to the database (20 380 sequences). The data was searched using Mascot with carboamidomethylation on cysteine residues and dimethylation on lysine residues as fixed modifications. N-terminal dimethylation on peptide N-termini was set as a variable modification. Samples incubated with trypsin were searched with a semi-Arg-C specificity while samples double digested (Trypsin + Glu-C) were searched with no-enzyme specificity. A MS mass tolerance of 7 ppm was used while a MSMS tolerance of 0.5 and 0.02 Da was used for CID and HCD data, respectively.

After database searching the peptides were filtered: only peptides with an FDR $\leq$ 0.01 (high-confident) were considered, in addition only peptides observed in two out of three technical replicates were considered. Furthermore, only peptides which were observed in both biological experiments were considered. The experimental variation of the data (HSP90 and PM) is shown in Supplementary Figure S1A.

N-terminal and dimethylated peptides were considered significantly regulated, and thus generated by proteolysis, if a protease/control ratio $\geq$ 2.5. Conversely, peptides with a protease/control ratio $\leq$ 0.4 were depleted by proteolysis. If a peptide was observed only in the CtsD treated samples, i.e., a singlet peak, a protease/control ratio of 100 was assigned.

The non-prime sites were matched to the corresponding prime sites identified by MS and a heat map was generated to determine whether the peptides (and their cleavage sites) identified in our experiment were in agreement with the known enzyme specificity of Cathepsin D.

a

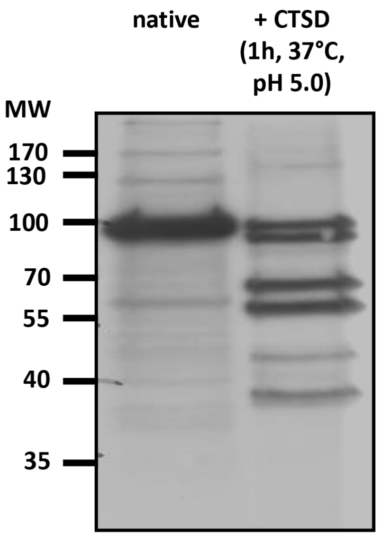

b

			BR1			BR2		
Start	End	Sequence	PSMs	Quan Count	Average	PSMs	Quan Count	Average
38	46	YSNKEIFLR	6	3	100	4	3	100
45	60	LRELISNSSDALDKIR	33	9	100	33	9	100
45	60	LRELISNSSDALDKIR*	9	3	100	14	5	100
46	60	RELISNSSDALDKIR	33	9	100	34	9	100
46	60	RELISNSSDALDKIR*	33	9	100	30	9	93.2
48	60	LISNSSDALDKIR	12	3	100	22	7	87.4
49	60	ISNSSDALDKIR	24	6	100	23	6	100
49	60	ISNSSDALDKIR*	12	5	100	15	6	100
77	87	HINLIPNKQDR	6	3	97.8	11	3	14.0
385	400	IRGVVDSEDLPLNISR*	14	4	100	19	6	100
388	401	VVDSEDLPLNISRE*	19	6	100	12	3	100
438	456	YEQFSKNIKLGIHEDSQNR	11	3	100	11	6	75.9
466	483	YTSASGDEMVSLKDYCTR	3	2	100	10	3	100
677	687	SLEDPQTHANR	16	4	94.5	6	2	31.0

c

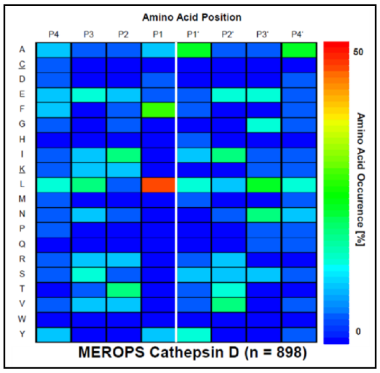

d

MPEETQTQDQPMEEEEVETFAFQAEIAQLMSLIINTF|YSNKEIF|L|RE|L|ISNSSD
ALDKIRYESLTDPSKLDSGKEL|HINLIPNKQDRTLTIVDTGIGMTKADLINNLGTIAKS
GTKAFMEALQAGADISMIGQFGVGFYSAYLVAEKVTVITKHNDDEQYAWESSAGG
SFTVRTDTGEPMGRGTKVILHLKEDQTEYLEERRIKEIVKKHSQFIGYPITLFVEKERD
KEVSDDEAEEKEDKEEEKEKEEKESEDKPEIEDVGSDEEEEKKDGDKKKKKKIKEKYID
QEELNKTKPIWTRNPDDITNEEYGEFYKSLTNDWEDHLAVKHFSVEGQLEFRALLFV
PRRAPFDLFENRKKKNNIKLYVRRVFIMDNCEELIPEYLNF|IRG|*VVDSEDLPLNISR*
*E*MLQQSKILKVIRKNLVKKCLELFTELAEDKENYKKF|YEQFSKNIKLGIHEDSQNRKK
LSELLRY|YTSASGDEMVSLKDYCTRMKENQKHIYYITGETKDQVANSAFVERLRKH
GLEVIYMIEPIDEYCVQQLKEFEGKTLVSVTKEGLELPEDEEEKKKQEEKKTKFENLCK
IMKDILEKKVEKVVVSNRLVTSPCCIVTSTYGWTANMERIMKAQALRDNSTMGYM
AAKKHLEINPDHSIIETLRQKAEADKNDKSVKDLVILLYETALLSSGF|SLEDPQTHAN
RIYRMIKLGLGIDEDDPTADDTSAAVTEEMPPLEGDDDTSRMEEVD

Supplementary Figure S1: Identification of the CtsD cleavage site within HSP90. a. Purified recombinant HSP90 has been treated with CtsD for the indicated time. The left lane shows the untreated, the right lane the CtsD treated HSP. The gel was stained with SYPRO ruby. **b.** Shows the n-terminal peptides identified by MS in the ATOMS approach from two independent biological replicates (BR1/BR2). **c.** Heatmap of CtsD cleavage sites based on the MEROPS database compared with the cleavage sites identified in this analysis. **d.** Amino acid sequence showing the identified peptides (red) and the cleavage sites (green line).

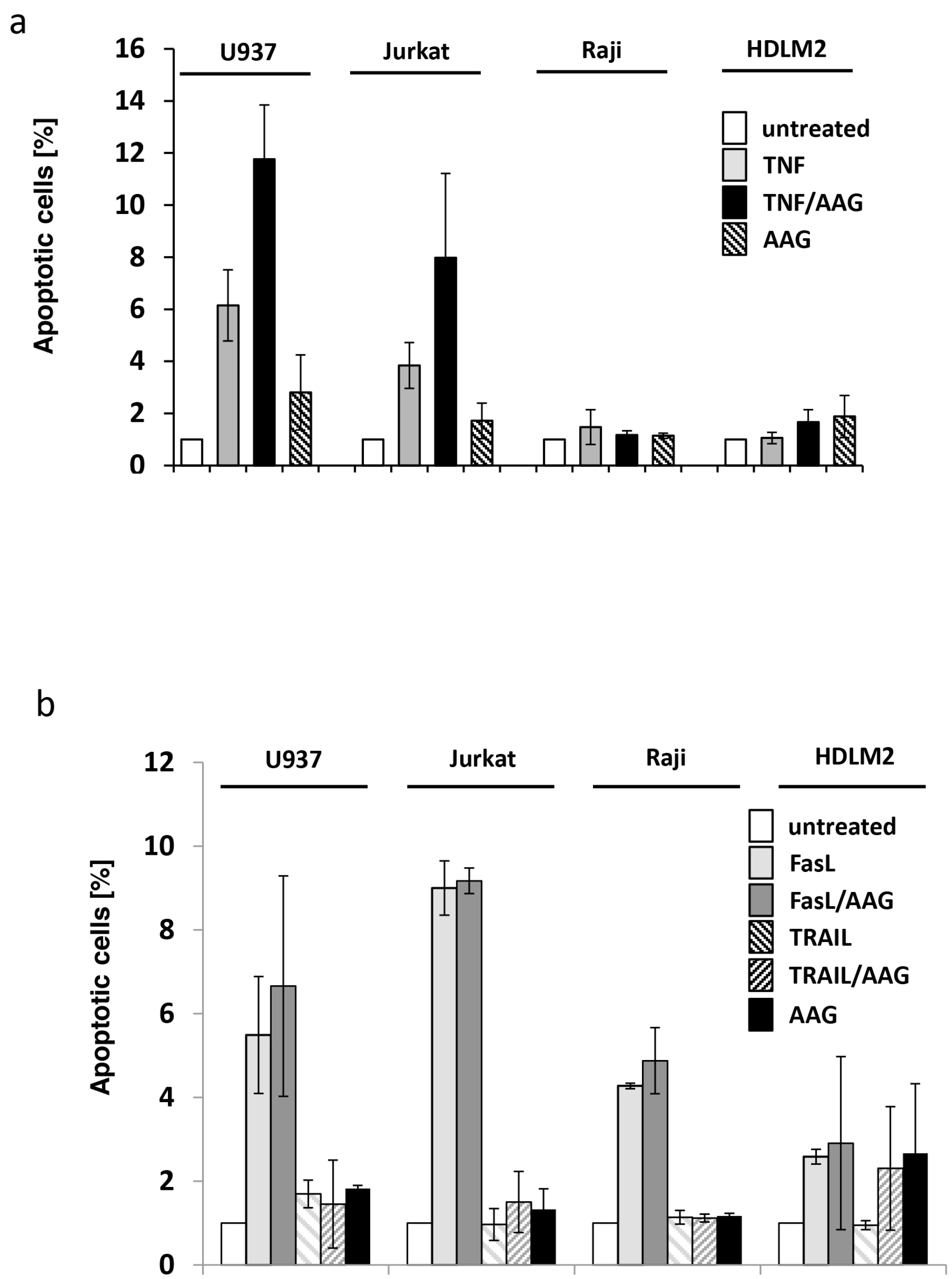

Supplementary Figure S2: Analysis of apoptosis induction in U937, Jurkat, Raji and HDLM2 cell lines. a. Cells were left untreated, TNF treated, TNF/17AAG treated or incubated with 17AAG alone. Potentiation of apoptosis occurred only in U937 and Jurkat cells. **b.** Cells were treated with FasL and TRAIL in combination or without 17-AAG. Co-Treatment did not result in enhanced apoptosis.
